# The Hampering Effect of Heavy Water (D_2_O) on Oscillatory Peptidization of Selected Proteinogenic α-Amino Acids

**DOI:** 10.3389/fchem.2020.00541

**Published:** 2020-06-30

**Authors:** Agnieszka Fulczyk, Eliza Łata, Ewa Talik, Teresa Kowalska, Mieczysław Sajewicz

**Affiliations:** ^1^Institute of Chemistry, University of Silesia, Katowice, Poland; ^2^Department of Physics of Crystals, University of Silesia, Katowice, Poland

**Keywords:** spontaneous oscillatory peptidization, proteinogenic α-amino acids, soluble peptides, insoluble peptides, D_2_O

## Abstract

We present an overview of our studies on the hampering effect of heavy water (D_2_O) on spontaneous oscillatory peptidization of selected proteinogenic α-amino acids. The investigated set of compounds included three endogenous and two exogenous species. The experiments were carried out with use of high-performance liquid chromatography (HPLC), mass spectrometry (MS) and scanning electron microscopy (SEM). These techniques were chosen to demonstrate spontaneous oscillatory peptidization of α-amino acids in an absence of D_2_O (HPLC) and the hampering effect of D_2_O on peptidization (HPLC, MS and SEM). The HPLC analyses were carried out at 21 ± 0.5°C with each α-amino acid freshly dissolved in the binary liquid mixture of organic solvent + H_2_O, 70:30 (v/v) or in pure D_2_O for several dozen hours or several hours, respectively. The analyses with use of MS and SEM were carried out, respectively, after 7 days and 1 month of sample storage period in the darkness at 21 ± 0.5°C and for these experiments, each α-amino acid was dissolved in the liquid mixture of organic solvent + X, 70:30 (v/v), where X: H_2_O + D_2_O in volume proportions from 30:0 to 0:30. The results obtained with use of HPLC, MS and SEM point out to the strong hampering effect of D_2_O on the oscillations and peptidization yields, yet the dynamics of these processes significantly depends on chemical structure of a given α-amino acid.

## Spontaneous Oscillatory Peptidization of Proteinogenic α-Amino Acids and Other Structurally Related Compounds

Our research on spontaneous oscillatory processes in organic chemistry has started with an observation that the thin-layer chromatographic (TLC) runs of ibuprofen resulted in an irreproducible retardation factor (*R*_F_) which was, however, confined to the two borderlines (Sajewicz et al., 2005a). Although ibuprofen is regarded as structurally stable and indestructible in the binary organic-aqueous solvents, irreproducibility of retardation factor (R_F_) was clearly related to the length of its storage period in solution. Later, an analogical phenomenon was observed with other profen drugs (Sajewicz et al., 2005b, 2006a,b, 2007; Marczak et al., 2006), α-hydroxy acids (Sajewicz et al., 2008a, 2009) and α-amino acids (Sajewicz et al., 2008b,c). Common structural denominator was that all these compounds were chiral low molecular weight carboxylic acids. Soon it became evident that the borderlines of the retardation factor (*R*_F_) values were those characterizing pure (+)- and pure (–)-enantiomer, and the phenomenon was the oscillatory chiral inversion (Sajewicz et al., 2008a,c, 2009).

Based on general knowledge of reaction mechanisms, it was understood that the elementary steps of chiral inversion proceed *via* the non-chiral intermediary products and their presence was demonstrated in the experiment performed with use of the high-performance liquid chromatography (HPLC) employing chiral stationary phase dedicated to the enantioseparation of α-amino acids (Sajewicz et al., 2014b). As the test analytes, two enantiomers of phenylglycine (Phg) were used and separation thereof was carried out for the period of 44 h. After 6 h, two separate chromatographic peaks of L-PhG and D-Phg coalesced into one and the coalescence period lasted 12 h. After that time, the single coalesced peak split to again give two peaks of L-PhG and D-Phg. This process was monitored with use of the DAD detector and a clear difference was observed between the identical UV spectra valid for L-PhG and D-Phg on the one hand, and the UV spectrum of the coalesced peak on the other. The latter one was ascribed to the non-chiral intermediary product(s) and its relative longevity (12 h) was regarded as striking and noteworthy. Our discovery of spontaneous oscillatory chiral inversion was approved by some other researchers as a reasonable justification of their own striking and quite unexpected results (e.g., Rincon et al., 2009; Stich et al., 2013).

Proteinogenic α-amino acids as the smallest “bricks” of all living matter seem the most important species undergoing oscillatory chiral inversion. Soon it became clear that α-amino acids undergo oscillatory peptidization as well. The main analytical tool to monitor this phenomenon was the non-chiral HPLC system and we focused on signals originating from the monomeric (i.e., non-peptidized) α-amino acids and their oscillatory concentration changes in the function of time, due to the peptidization–depeptidization process. We published a number of reports on this phenomenon which was also theoretically modeled, and the most important papers are (Sajewicz et al., 2010, 2014a; Godziek et al., 2016; Maciejowska et al., 2016). Up to our best knowledge, there had never been any earlier report neither on spontaneous peptidization of α-amino acids under such mild external conditions (dissolution at ambient temperature in an organic aqueous solvent), nor on oscillatory nature of this process. All our investigations were inspired by an interest in chemical evolution preceding biological one, in which spontaneous peptidization of α-amino acids might have played a significant role.

## D_2_O in Biochemical Studies and the Aim of Our Research Project

With an improved availability of D_2_O in the thirties of the twentieth century, its influence on animals and plants has become investigated and a slowdown effect on life processes was recognized, in extreme cases resulting in an organism's death (Harvey, 1934; Lewis, 1934; Katz et al., 1962). Current experiments with D_2_O largely focus on its apoptotic effect on cancer cells (Takeda et al., 1998; Hartmann et al., 2005). All these results instigated our interest in an impact of D_2_O on the dynamics of spontaneous oscillatory peptidization and peptidization yields with selected endogenous (L-Cys, L-Pro, and L-Ala) and exogenous (L-Met and L-Hyp) α-amino acids as the smallest building blocks of all living matter.

## Effect of D_2_O on Spontaneous Oscillatory Peptidization

Five α-amino acids (L-Cys, L-Met, L-Pro, L-Hyp, and L-Ala) were dissolved in the binary liquid mixture of the organic solvent + H_2_O, 70:30 (v/v) (where organic solvent: acetonitrile or methanol) and due to oscillatory peptidization-depeptidization process, the monomer concentrations of these compounds underwent spontaneous oscillatory changes. These changes were recorded with use of the non-chiral HPLC and graphically presented in supplementary materials of papers (Fulczyk et al., 2018, 2019a,b, 2020a,b) as time series, together with the corresponding plots of the Fourier-transformed data. From Fourier transformation it came out that with two endogenous species (L-Cys and L-Pro) the circadian rhythm of the oscillatory concentration changes occurs as equal to ca. 24 and ca. 20.8 h, respectively, and with two exogenous species (L-Met and L-Hyp) no periodicity of oscillations is observed. With the third endogenous species (L-Ala), the oscillatory pattern is still different. After two initial and not periodic oscillations lasting for ca.10 h, the investigated system reaches a steady state.

Dissolution of the investigated α-amino acids in pure D_2_O results in full inhibition of oscillatory peptidization, as confirmed by HPLC. Differentiation of peptidization dynamics and peptide yields becomes visible only, when proportions of D_2_O in solution stepwise change. Mass spectrometry (MS) and scanning electron microscopy (SEM) were used to record differences in the hampering effect of D_2_O on dynamics of peptidization and peptide yields depending of proportions of heavy water in solution.

**Figure 1:**
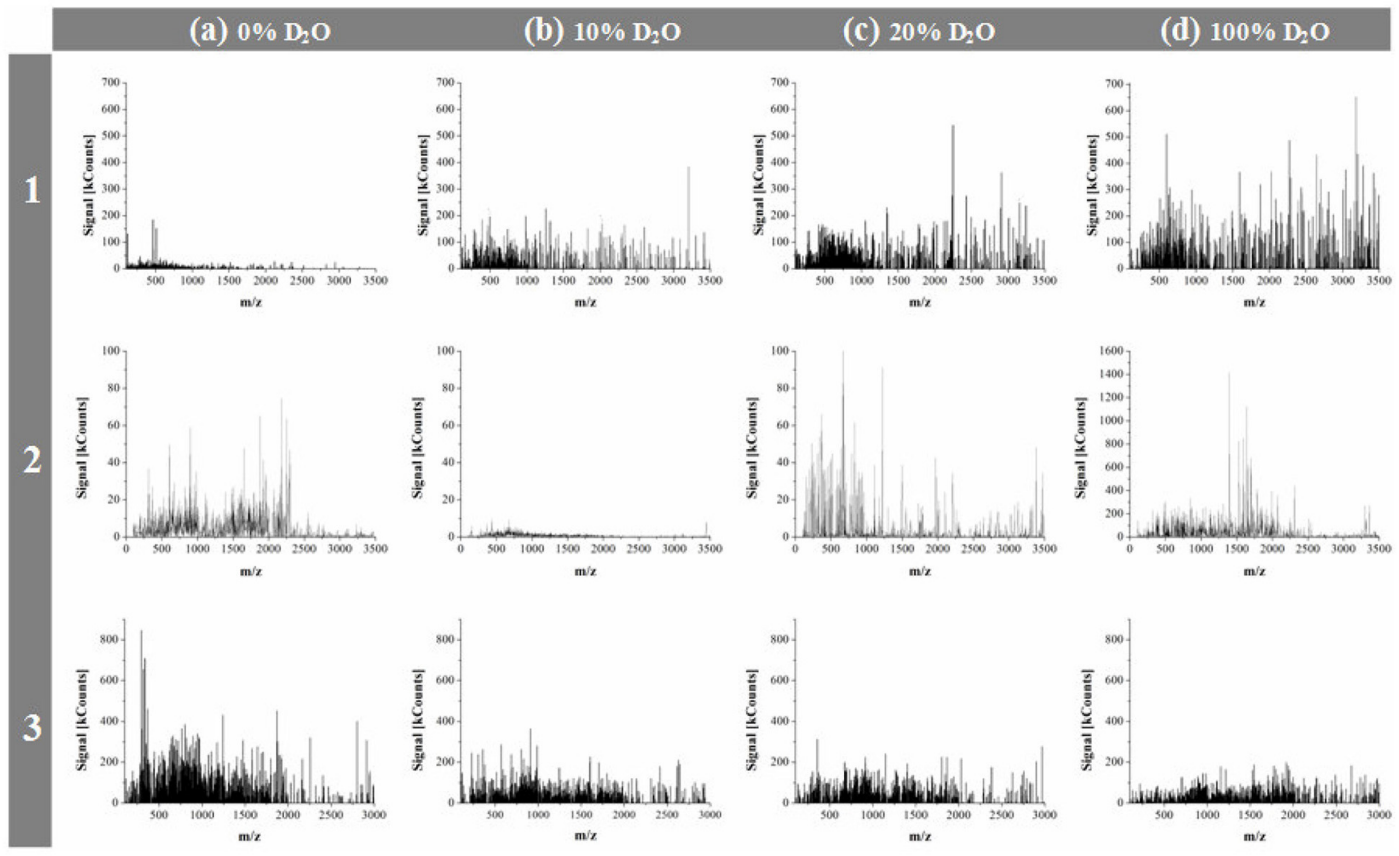
Mass spectra recorded for L-Cys (1), L-Pro (2), and L-Ala (3), respectively, dissolved in organic solvent + X, 70:30 (v/v), where X: the binary mixture of H_2_O + D_2_O in the changing volume proportions; **(a)** 0% D_2_O; **(b)** 10% D_2_O; **(c)** 20% D_2_O, and **(d)** 100% D_2_O. Organic solvent: ACN (L-Cys), or MeOH (L-Pro and L-Ala); [adapted from Figures in Fulczyk et al. (2018, 2019a, 2020a)].

Apart from considering the investigated compounds as belonging to two groups of endogenous and exogenous α-amino acids, they can also be viewed as three structural entities: two sulfur atom containing species (L-Cys, L-Met), two pyrrolidine ring and secondary amino group containing species (L-Pro, L-Hyp), and the simplest endogenous α-amino acid, L-Ala. First, let us reflect on the mass spectrometric results. As mass spectrometric technique used in this study was applicable to monitoring liquid samples alone, the obtained mass spectra provided information on changing contents of soluble lower molecular weight peptides depending on proportions of D_2_O in solution. For each α-amino acid considered, mass spectra were recorded after 7 days sample storage period in the darkness at 21 ± 0.5°C, in presence of 0, 5, 10, 20, 30, and 100% D_2_O. The obtained MS results were originally published in papers (Fulczyk et al., 2018, 2019a,b, 2020a,b). It was shown that with increasing proportions of D_2_O in solution, L-Cys and L-Met produce growing yields of soluble lower peptides. With L-Pro, the yields of soluble lower peptides are decreasing up to 10% D_2_O, but then with higher amounts of D_2_O these yields start gradually growing. With L-Hyp and L-Ala, the decreasing yields of soluble lower peptides are observed in pace with growing proportions of D_2_O in solution. It can be concluded that the response of five investigated α-amino acids to changing quantitative proportions of D_2_O in solution depends on chemical structure of individual species rather than on belongingness to the endogenous/exogenous category, or on structural similarity of species (although a similar trend is observed with two sulfur atom containing α-amino acids, i.e., L-Cys and L-Met). Figure 1 briefly illustrates an impact of selected proportions of D_2_O on the yields of soluble lower peptides upon the examples of L-Cys, L-Pro, and L-Ala.

**Figure 2:**
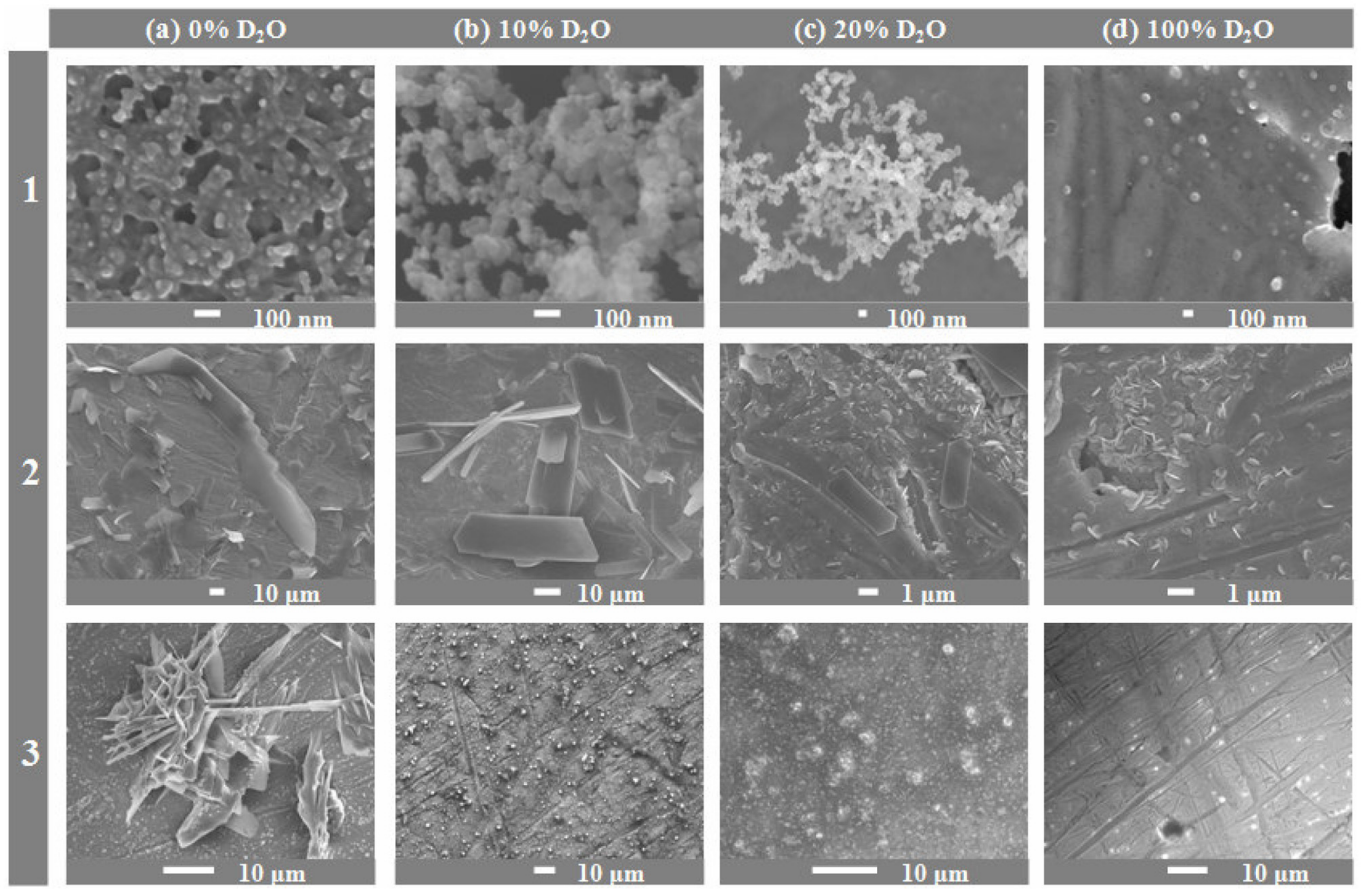
Scanning electron micrographs recorded for L-Cys (1), L-Met (2), and L-Pro (3), respectively, dissolved in organic solvent + X, 70:30 (v/v), where X: the binary mixture of H_2_O + D_2_O in the changing volume proportions; **(a)** 0% D_2_O; **(b)** 10% D_2_O; **(c)** 20% D_2_O, and **(d)** 100% D_2_O. Organic solvent: ACN (L-Cys and L-Met), or MeOH (L-Pro). Magnifications of individual micrographs: (a_1_) × 100,000; (a_2_) × 550; (a_3_) × 1,900; (b_1_) × 100,000; (b_2_) × 1,000; (b_3_) × 750; (c_1_) × 30,000; (c_2_) × 7,500; (c_3_) × 2,500, (d_1_) × 37,000; (d_2_) × 10,000; (d_3_) × 1,500 [partially adapted from Figures in Fulczyk et al. (2018, 2019a,b)].

Now let us consider the results originating from the scanning electron microscopy. For each α-amino acid under the discussion, scanning electron micrographs were recorded after 1 month sample storage period in the darkness at 21 ± 0.5°C, in presence of 0, 5, 10, 20, 30, and 100% D_2_O. As this technique was applicable to solid samples alone, basic information which can be derived from the obtained micrographs focuses on changing yields of insoluble higher peptides, depending on proportions of D_2_O in solution. The obtained results unequivocally demonstrate that irrespective of the α-amino acid considered, in the presence of D_2_O in solution the hampering effect on peptidization process is observed and consequently, the yields of higher insoluble peptide drastically lower. Apparently, the higher is the D_2_O proportion in solution, the stronger pronounced is the hampering effect. For each species, a series of photographs was taken at different magnifications to best illustrate regularities and trends of this inhibitory effect and they were published in papers (Fulczyk et al., 2018, 2019a,b, 2020a,b). In Figure 2, we present selected micrographs valid for L-Cys, L-Met, and L-Pro, which illustrate an impact of the growing proportions of D_2_O on formation of insoluble higher peptides. They also allow a comparison of different structural forms of peptides derived from each individual α-amino acid. Peptides derived from L-Cys are spherical and gather in greater and spongy-looking structures. Peptides derived from L-Met are the flat, elongated laminas with sharp edges, and peptides derived from L-Pro resemble the 3D starry-looking objects of different sizes. All these micrograph series collected in Figure 2 provide convincing evidence that the increasing proportions of D_2_O in solutions really result in diminishing yields of higher insoluble peptides.

## Conclusions

The starting point of our research project was demonstration with use of the non-chiral HPLC that all investigated proteinogenic (endogenous and exogenous) α-amino acids dissolved in an organic-aqueous solvent yet in an absence of D_2_O undergo spontaneous oscillatory peptidization. Then we focused on the impact of D_2_O on spontaneous oscillatory peptidization process carried out either in pure heavy water, or in the mixture of organic solvent, H_2_O and D_2_O. With use of scanning electron microscopy (SEM), it was shown that D_2_O hampers formation of insoluble higher peptides with all investigated species and this effect is monotonously dependent on proportions of D_2_O in solution. With use of mass spectrometry (MS), it was shown though that D_2_O affects formation of the soluble lower peptides in a less straightforward manner. With L-Cys and L-Met, growing proportions of D_2_O in solution result in growing yields of respective lower soluble peptides. With L-Hyp and L-Ala, growing proportions of D_2_O in solution result in declining yields of respective peptides. Response of L-Pro to proportions of D_2_O in solution is non-monotonous. Initially, growing proportions of D_2_O in solution result in the declining yields of the lower soluble peptides, but for the D_2_O proportions above 10% these yields rather unexpectedly start growing. Within the framework of the adopted research project, we disclosed individual patterns of spontaneous oscillatory peptidization in an absence of heavy water and also inhibition of this process with D_2_O under the assumed working conditions for three endogenous and two exogenous α-amino acids.

Summing up, even if the research perspective outlined in this study is currently absent from consciousness of scientific community worldwide or underestimated by many (partially due to serious limitations of effective analytical tools), we firmly believe in purposefulness of carrying out similar research even on a much larger scale, simply because we can see its relevance to a wide spectrum of diverse scientific disciplines spanned between evolutionary history of life and contemporary medicine.

## Data Availability Statement

The original contributions presented in the study are included in the article/supplementary materials, further inquiries can be directed to the corresponding author/s.

## Author Contributions

All authors equally contributed to preparation of the submitted manuscript.

## Conflict of Interest

The authors declare that the research was conducted in the absence of any commercial or financial relationships that could be construed as a potential conflict of interest.
